# Validation of the Passive Surveillance Stroke Severity score in three Canadian provinces

**DOI:** 10.23889/ijpds.v9i5.2732

**Published:** 2024-09-18

**Authors:** Alison L. Park, Sandra Peterson, Yinshan Zhao, Peter C. Austin, Jiming Fang, Michael D. Hill, Noreen Kamal, Thalia S. Field, Raed A. Joundi, Moira K. Kapral, Amy Y. X. Yu

**Affiliations:** 1 ICES; 2 Centre for Health Services and Policy Research, University of British Columbia; 3 Population Data BC, University of British Columbia; 4 Departments of Clinical Neurosciences, Community Health Sciences, Medicine, Radiology and Hotchkiss Brain Institute, University of Calgary; 5 Department of Industrial Engineering, Dalhousie University; 6 Department of Medicine (Neurology), Vancouver Stroke Program, University of British Columbia; 7 Department of Medicine, Hamilton Health Sciences Centre, McMaster University; 8 Department of Medicine (General Internal Medicine), University of Toronto-University Health Network; 9 Department of Medicine (Neurology), University of Toronto, Sunnybrook Health Sciences Centre

## Objective

Adjusting for stroke severity is critical in stroke outcomes research. The Passive Surveillance Stroke SeVerity (PaSSV) score is an administrative data-based measure of stroke severity, initially derived in Ontario, Canada using data between 2002-2013. We assessed its geographical and temporal external validity in British Columbia (BC), Nova Scotia (NS), and Ontario, Canada.

## Approach

In each province, we identified adult in-patients with ischemic stroke or intracerebral hemorrhage and admitted from an emergency department between 2014-2019 and calculated their PaSSV score using linked administrative data. We used Cox proportional hazards models to evaluate the association between the PaSSV score and the hazard of death over 30 days and the cause-specific hazard of admission to long-term care over 365 days. We assessed the models’ discriminative values using Uno’s c-statistic, comparing models with versus without PaSSV.

## Results

We included 86,142 patients (n=18,387 in BC, n=65,082 in Ontario, n=2,673 in NS). The mean and median PaSSV were similar across provinces. Higher PaSSV score, reflecting lower stroke severity, was associated with a lower mortality (hazard ratio and 95% confidence intervals 0.70 [0.68-0.71] in BC, 0.69 [0.68-0.69] in Ontario, 0.72 [0.68-0.75] in NS) and long-term care admission (0.77 [0.76-0.79] in BC, 0.84 [0.83-0.85] in Ontario, 0.86 [0.79-0.93] in NS). Including PaSSV in the multivariable models improved model fit according to the c-statistics.

## Conclusion

We showed that PaSSV has geographical and temporal validity. It is a useful tool for risk-adjustment in multi-jurisdiction stroke outcomes research, and a valuable addition to be included in the national algorithm inventory.

